# Transcriptomic-Based Quantification of the Epithelial-Hybrid-Mesenchymal Spectrum across Biological Contexts

**DOI:** 10.3390/biom12010029

**Published:** 2021-12-25

**Authors:** Susmita Mandal, Tanishq Tejaswi, Rohini Janivara, Syamanthak Srikrishnan, Pradipti Thakur, Sarthak Sahoo, Priyanka Chakraborty, Sukhwinder Singh Sohal, Herbert Levine, Jason T. George, Mohit Kumar Jolly

**Affiliations:** 1 Centre for BioSystems Science and Engineering, Indian Institute of Science, Bangalore 560012, India; susmitam@iisc.ac.in (S.M.); tanishqt@iisc.ac.in (T.T.); sarthaksahoo@iisc.ac.in (S.S.); priyanka08993@gmail.com (P.C.); 2 Undergraduate Programme, Indian Institute of Science, Bangalore 560012, India; 3 Department of Biological Sciences, Georgia Institute of Technology, Atlanta, GA 30332, USA; rohini97js@gmail.com; 4 Department of Biotechnology, Indian Institute of Technology, Kharagpur 721302, India; syamanthak.s@gmail.com (S.S.); pradiptithakur.iitkgp@gmail.com (P.T.); 5 Respiratory Translational Research Group, Department of Laboratory Medicine, School of Health Sciences, College of Health and Medicine, University of Tasmania, Launceston 7248, Australia; sukhwinder.sohal@utas.edu.au; 6 Departments of Physics and Bioengineering, Northeastern University, Boston, MA 02115, USA; h.levine@northeastern.edu; 7 Center for Theoretical Biological Physics, Rice University, Houston, TX 77030, USA

**Keywords:** EMT scoring, hybrid epithelial/mesenchymal, single-cell RNA-seq, cellular reprogramming

## Abstract

Epithelial-mesenchymal plasticity (EMP) underlies embryonic development, wound healing, and cancer metastasis and fibrosis. Cancer cells exhibiting EMP often have more aggressive behavior, characterized by drug resistance, and tumor-initiating and immuno-evasive traits. Thus, the EMP status of cancer cells can be a critical indicator of patient prognosis. Here, we compare three distinct transcriptomic-based metrics—each derived using a different gene list and algorithm—that quantify the EMP spectrum. Our results for over 80 cancer-related RNA-seq datasets reveal a high degree of concordance among these metrics in quantifying the extent of EMP. Moreover, each metric, despite being trained on cancer expression profiles, recapitulates the expected changes in EMP scores for non-cancer contexts such as lung fibrosis and cellular reprogramming into induced pluripotent stem cells. Thus, we offer a scoring platform to quantify the extent of EMP in vitro and in vivo for diverse biological applications including cancer.

## 1. Introduction

Epithelial-Mesenchymal Plasticity (EMP) is an important feature of cancer metastasis and therapy resistance, the two major clinical challenges that claim the majority of cancer-related deaths [1]. EMP involves dynamic and reversible switching among multiple phenotypes along the epithelial-hybrid-mesenchymal spectrum. It encompasses both EMT (Epithelial-to-Mesenchymal Transition) and MET (Mesenchymal-to-Epithelial Transition). Originally considered to be binary transitions, EMT and MET are both now understood as multi-step processes, and cells can execute these programs to varying degrees, enabling one or more hybrid epithelial/mesenchymal (E/M) phenotypes [2,3,4]. EMP usually entails changes in cell–cell adhesion, migration, and invasion; while EMT is often involved with cells escaping the primary tumor and initiating metastasis, MET is thought to be important for colonization, the last step of metastasis. Besides these features, EMP is also implicated in conferring tumor-initiation potential [5,6], immune evasion [7,8,9], and resistance to various chemotherapeutic drugs and targeted therapies [10,11,12]. Thus, EMP can be considered as the “motor of cellular plasticity” [13], which enhances cancer cell fitness in a variety of biological contexts.

Recent preclinical and clinical observations have suggested the high metastatic potential of hybrid E/M phenotypes, and their association with worse patient survival across cancer types [14,15,16,17,18,19,20,21]. Hybrid E/M phenotypes have also been observed in circulating tumor cells (CTCs); their higher frequency is often concomitant with worse clinicopathological features [22,23,24,25]. The ability of hybrid E/M cells to form clusters of CTCs can also escalate their metastatic fitness, given the disproportionately high metastatic burden of CTC clusters [26,27,28]. Given the context-specific diversity of hybrid E/M phenotypes [29], it is imperative that EMP be quantified as a continuum or spectrum, through integrating various experimental and/or computational methods.

At the transcriptomic level, various computational methods have been proposed to quantify the EMP spectrum by calculating the extent to which a given sample has undergone EMT/MET (hereafter, referred to as the “EMT score”). First, using gene expression from non-small cell lung cancer (NSCLC) cell lines and patients, a 76-gene EMT signature was identified and then used to derive a score (hereafter, referred to as the “76GS score”) based on the relative enrichment of expression levels of epithelial-associated genes. The 76GS score is a weighted sum of the expression levels of these 76 genes, with the weight being the correlation coefficient of the corresponding gene with that of E-cadherin (CDH1) levels in the given dataset. Thus, the higher the 76GS score is, the more epithelial a given sample is [30,31]. Second, a two-sample Kolmogorov–Smirnov method was used to calculate a score (hereafter, referred to as the “KS score”) on the interval [-1, +1] to depict the EMP status of cell lines and tumors using a set of around 300 genes containing epithelial and mesenchymal associated ones. The higher the KS score is, the more mesenchymal a sample is [32]. Third, a multinomial logistic regression method implemented on NCI-60 expression data quantified the extent of EMT on the interval [0,2] (hereafter, referred to as the “MLR score”) by calculating the probabilities for a given sample to belong to E, M, or hybrid E/M states [33]. Three predictor genes, together with a few normalizers, are used to predict the EMT status using the MLR metric. Higher MLR scores depict a relatively enriched mesenchymal phenotype. Notably, all these scoring metrics have been originally trained on microarray datasets.

A previous study compared these three methods—each of which utilizes a distinct gene list and algorithm—and observed that these methods were largely well-correlated with one another in terms of quantifying EMP across multiple microarray datasets [34]. This analysis suggested that the EMT scores were concordant among one another, i.e., the 76GS scores correlated negatively with their MLR and KS counterparts, both of which positively correlated with one another (as the higher the 76GS scores are, the more epithelial the samples are, whereas in the case of KS and MLR, it is the opposite, the higher the scores are, the more mesenchymal the samples are). However, the following two key questions remain unanswered: (a) can all three of these scoring metrics quantify the EMP spectrum for bulk and single-cell RNA-seq data with the same level of consistency? (b) can these scores, all constructed on cancer-related datasets, be helpful in estimating the extent of EMP in non-cancer scenarios as well?

Here, we have addressed these limitations by analyzing multiple bulk and single-cell RNA-seq datasets, as well as investigating both microarray and RNA-seq datasets for the following two non-cancer cases where EMP has been reported: (a) lung diseases—chronic obstructive pulmonary disease (COPD) and fibrosis [35,36] and (b) reprogramming induced pluripotent stem cells (iPSCs) [37]. We demonstrate consistency amongst the EMT scoring metrics in quantifying the EMP spectrum across these biological contexts, as well as the heterogeneity of EMP phenotypes in single-cell RNA-seq datasets. Finally, through a pan-cancer analysis of RNA-seq data available via The Cancer Genome Atlas (TCGA), we show that the association of EMP with patient survival is context-specific. Despite using diverse gene-sets and methodology to quantify EMP, a convergence of these three methods suggests commonalities in the different trajectories that cells undergoing EMT/MET can take in a high-dimensional landscape. Moreover, our results offer proof-of-principle that these metrics, all of which were derived based on cancer, can successfully quantify EMP in other useful non-cancer biological contexts too.

## 2. Materials and Methods

### 2.1. Software and Datasets

We downloaded high-throughput transcriptomics data (bulk and single cell) from GEO and EMBL-EBI databases. Microarray datasets were downloaded using GEOquery R Bioconductor package [38]. TCGA expression and survival data were obtained from the UCSC Xena browser (https://xena.ucsc.edu/) (accessed on 18 November 2020). Statistical analysis, survival analysis and plots were all conducted in R version 4.0.3. Function *ggplot* was used for plots.A list of all softwares used and their URL is given in Table S9.

### 2.2. Preprocessing of Datasets

After downloading the HTS datasets, a quality check was performed using FASTQC [39] (https://www.bioinformatics.babraham.ac.uk/projects/fastqc/, accessed on 18 November 2020). Bulk and single-cell RNA seq data were aligned to reference genome (hg38/mm10, appropriately) using STAR- aligner [40]. Samtools [41] was used to modify alignment files (SAM/BAM), and htseq-count [42] was used to calculated the read counts. Using these read counts, TPM expression was calculated using custom scripts and log2 normalized TPM values were used for calculating the EMT scores. In the case of microarray datasets, they were preprocessed to obtain the gene-wise expression for each sample from probe-wise expression matrix. If there were multiple probes mapping to one gene, then the mean expression of all mapped probes was considered for that gene.

### 2.3. T-Test

Two-tailed Student’s *t*-test with unequal variance was performed to compare between samples in many bar plots. Error bars denoted the standard deviation (statistical significance at *p* < 0.05).

### 2.4. Calculation of EMT Scores

EMT scores were calculated using all three methods—76GS, KS, and MLR—as previously conducted for microarray datasets [34]. MLR method, which was designed for microarray datasets [33], was adjusted to work for HTS transcriptomics data as well. The KS method has two gene lists—one for cell line, one for tumor. The corresponding gene list was applied, depending on the dataset. A list of genes common among the different scoring metrics is given in Table S8.

### 2.5. MLR Model Applied to RNA-Seq

We adapted a previously developed method of quantifying EMT spectrum trained on and designed to predict microarray samples [33]. Briefly, VIM, CDH1, and CLDN7 transcripts were identified to maximally predict NCI-60 holdout samples in a leave-one-out assessment utilizing two-dimensional multinomial logistic regression (MLR). These, together with a list of 20 normalizers, enable the assignment of each input (CLDN7, VIM/CDH1) to an ordered triple (P_E_, P_E/M_, P_M_) that characterizes the probability that a signature belongs either to the Epithelial (E), Mesenchymal (M), or hybrid (E/M) group. This ordered triplet is then projected onto the interval [0,2] with 0 designating a fully epithelial signature, 1 maximally hybrid signature, and 2 fully mesenchymal signature.

To apply the microarray based MLR model to RNA-seq data, we utilized transcriptomic data available in both formats on biological replicates [43]. Using the data available in Figure 2 (2 biological replicates for each of 6 time points), we restricted our analysis to the intersection of microarray and RNA-seq transcripts for genes represented in positive abundance for both datasets. Linear regression to the average of each biological replicate, producing a total of 6 slope-intercept pairs:

(1) $$y_{FPKM\to µArray}=\left[x_{FPKM}\;1\right]\left[\begin{array}{cccccc} 1.17 & 1.19 & 1.21 & 1.22 & 1.22 & 1.20\\ -6.82 & -7.04 & -7.26 & -7.31 & -7.10 & -6.93 \end{array}\right]$$

(2) $$y_{TPM\to µArray}=\left[x_{TPM}\;1\right]\left[\begin{array}{cccccc} 1.16 & 1.19 & 1.21 & 1.23 & 1.24 & 1.20\\ -6.83 & -7.04 & -7.26 & -7.31 & -7.10 & -6.93 \end{array}\right]$$

which were then averaged to be used as the fit parameters for cross-platform assessment:

(3) $${\bar{y}}_{FPKM}=\left[{\bar{x}}_{µArray}\;1\right]\left[\begin{array}{c} 1.21\\ -7.08 \end{array}\right]=\left[{\bar{x}}_{µArray}\;1\right]\left[\begin{array}{c} m_{FPKM}\\ b_{FPKM} \end{array}\right]$$

(4) $${\bar{y}}_{TPM}=\left[{\bar{x}}_{µArray}\;1\right]\left[\begin{array}{c} 1.21\\ -7.08 \end{array}\right]=\left[{\bar{x}}_{µArray}\;1\right]\left[\begin{array}{c} m_{TPM}\\ b_{TPM} \end{array}\right]$$

From these, unique microarray values, $x_{µArray}$, representative of RNA-seq values, may be calculated by the following inversion:

(5) $$x_{µArray}=\left(y_{RNASeq}-b_{RNAseq}\right)/m_{RNASeq}.$$

### 2.6. Survival Analysis

Different metrics of survival data were obtained from TCGA cohort. All samples were divided into 76GS^high^ and 76GS^low^, MLR^high^ and MLR^low^, KS^high^ and KS^low^ groups based on the mean (or median) of the respective scores of the samples. Kaplan–Meier analysis was performed using R package “survival” and plotted using R package “ggfortify”. A log rank test was used to calculate the *p*-values. The reported hazard ratio (HR) and confidence interval (95% CI) were estimated using cox regression.

### 2.7. ssGSEA

Single-sample GSEA (ssGSEA), an extension of Gene Set Enrichment Analysis (GSEA), calculates separate enrichment scores for each pairing of a sample and gene set. Each ssGSEA enrichment score represents the degree to which the genes in a particular gene set are coordinately up- or down-regulated within a sample. We used “HALLMARK_EMT” gene set from The Molecular Signatures Database (MSigDB) database and the scores were calculated using R package “ssgsea”.

## 3. Results

### 3.1. EMT Scoring Methods Show Concordant Trends across Bulk RNA-Seq Datasets

We used the three different EMT scoring methods—76GS, KS, and MLR—to quantify the extent of EMP in multiple RNA-seq datasets, as was previously conducted for microarray datasets [34]. Each method utilizes a distinct gene signature and underlying algorithm to compute an EMT score. The 76GS score is a weighted sum of the expression of 76 genes, where the weight factor is the correlation coefficient of that gene with the expression levels of CDH1 (E-cadherin), a canonical epithelial marker. Thus, a higher 76GS score corresponds to a more epithelial sample [30,31]. The KS scoring method compares the empirical distribution function to the cumulative distribution function for epithelial and mesenchymal signatures identified in cell lines and tumors. The KS score is constructed by taking the maximal difference in these distributions for each predictor, followed by normalization by the number of predictors, thus taking values between −1 and +1. Positive (resp. negative) KS scores correspond to a relative enrichment of the mesenchymal (resp. epithelial) signature [32]. The Multinomial Logistic Regression-based (MLR) method quantifies the extent of EMT on a scale of 0–2. MLR scores are calculated based on the probability of a given sample being assigned to the E, E/M, and M phenotypes. Thus, the higher the score is, the more mesenchymal the sample is [33]. While KS and 76GS methods operate on gene lists and can therefore be directly applied to both microarray and RNA-seq data, the MLR method utilizes the NCI-60 microarray data as a training set for regression. Therefore, applying these methods for analyzing RNA-seq data needs further customization, especially the MLR method.

**Figure 1:**
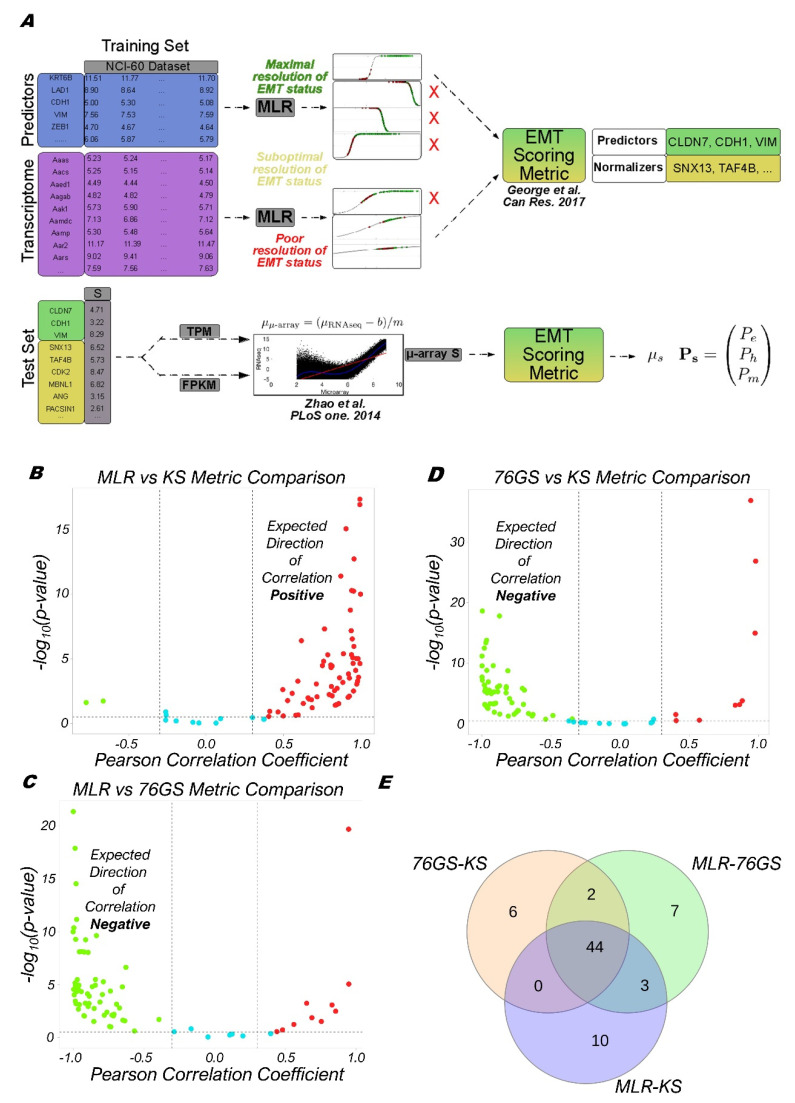
Schematic of the interconversion of MLR microarray and MLR RNA-seq scores and pairwise correlation plots of the three EMT scoring metrics. (**A**) Adaptation of microarray-based MLR approach to predict RNA-seq-based input. Predictors (resp. normalizers) in the NCI-60 microarray training set were selected as performed previously, based on their ability (resp. inability) to resolve the EMT status of the training set. Poor candidates (red X) in each case were omitted from inclusion in the proctor and normalizer set. Histograms (teal bars) and corresponding spline interpolate density function estimates (black curves). Linear regression between RNA-seq and microarray data given in Zhao et al. 2014 was used to relate a unique value of RNA-seq signal to the microarray-based model, conducted for both FPKM and TPM values. (**B**–**D**) Plots showing the pairwise correlation of EMT scoring metrics across 77 bulk RNA-Seq datasets, and for each sample –log_10_ (*p*-value) is plotted as a function of Pearson’s correlation coefficient. Thresholds considered for correlation (*R* < −0.3 (green points) or *R* > 0.3 (red points); vertical dashed grey lines) and *p*-values (*p* < 0.05; horizontal dashed grey lines) are denoted. Cyan points represent cases for which R > −0.3 and R < 0.3. (**E**) Venn diagram depicting the overlap of pairwise and full concordance for datasets that are significantly correlated in the expected direction.

We extended the previous MLR framework trained on the microarray-based transcriptomics of NCI-60 series to impute log_2_-normalized FPKM (Fragments Per Kilobase of transcript per million Mapped reads) or TPM (Transcripts Per Million) RNA-seq data. To achieve this, the log_2_ RNA-seq values for three predictors (CLDN7 (claudin 7), VIM (Vimentin), CDH1 (E-cadherin)) and 20 normalizers were linearly mapped to their corresponding microarray values (Figure 1A). This mapping was estimated for both FPKM- and TPM-normalized data by averaging over 24 previously published samples [43] where log_2_ microarray and log_2_ RNA-seq expression signatures were simultaneously available (Figures S1 and S2). The output of the updated MLR approach assigns a numerical EMT score, *S*, on the scale of [0,2] based on the probability of a sample’s categorization into one of the following three groups: E, E/M and M.

To check the concordance of these three EMT scoring metrics, we calculated the 76GS, KS, and MLR scores for 77 bulk high-throughput transcriptomics datasets. As expected, we found the 76GS scores to be negatively correlated (*r* < −0.3; *p* < 0.05) with the MLR and KS scores and found a positive correlation (*r* > 0.3; *p* < 0.05) between the MLR and KS scores across most datasets that contain cell lines and primary tumors across cancer types (Figure 1B–D; Table S1). A total of 44 out of the 77 (57.14%) datasets showed all three trends significantly (KS vs. MLR, MLR vs. 76GS, and 76GS vs. KS). Additionally, 52 (67.53%) cases exhibit the expected trends for 76GS vs. KS, similar to those seen for MLR vs. 76GS-56 (72.72%), and for MLR vs. KS-57 (74.02%) (Figure 1E). Furthermore, 72 datasets (93.5%) show concordance in the calculated EMT status/score in at least two of the three metrics. Thus, the MLR, 76GS, and KS scoring metrics show strong concordance among themselves for these 77 datasets.

**Figure 2:**
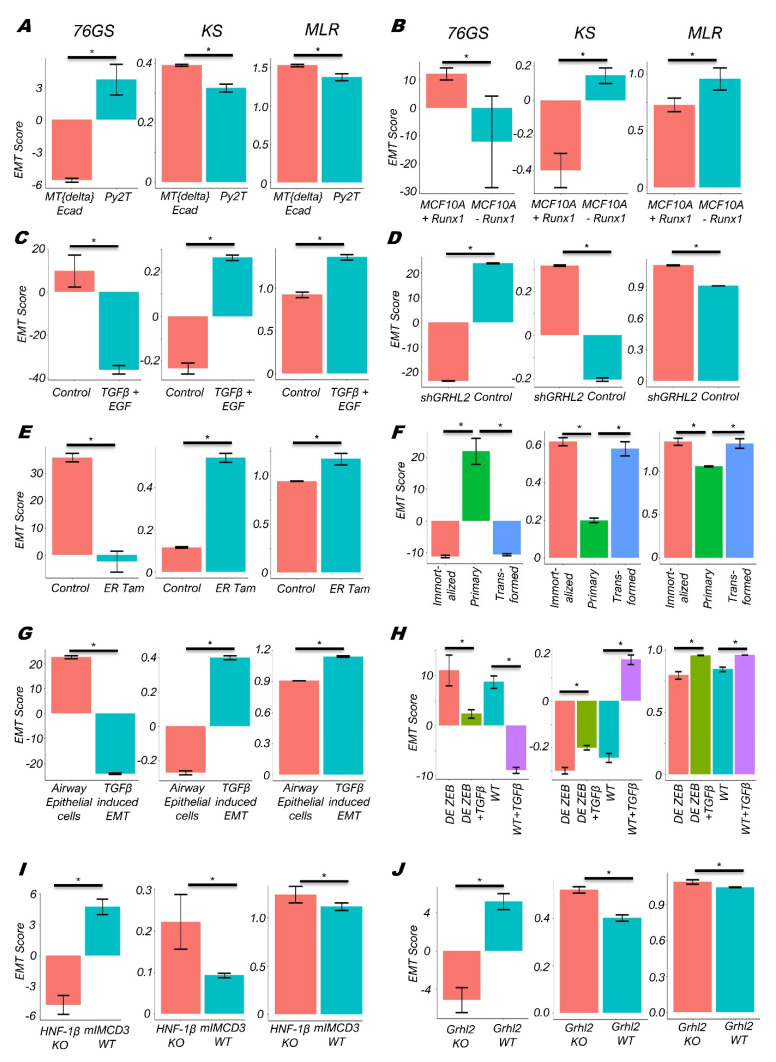
KS, 76GS, and MLR scores for different EMT associated datasets. (**A**) Py2T long term cells and mesenchymal breast cancer cells (MTΔECad) treated with different inhibitors (GSE118612). (**B**) MCF10A cells with or without depletion of Runx1 (GSE85857). (**C**) EMT induction in Hela cells by TGF-β + EGF (GSE72419) treatment. (**D**) EMT induction in small airway epithelial cells by TGF-β (GSE61220). (**E**) MCF10A cells with/without ZEB1 knocked out (KO) untreated or treated with TGF-β (GSE124843). (**F**) OVCA4209 cells with/without GRHL2 knockdown (GSE118407). (**G**) Grhl2 KO and wild type (WT) mice (GSE106130). (**H**) Primary, pre-malignant immortalized, and Ras-transformed human mammary epithelial cells (GSE110677). (**I**) EMT induction in HMLE/Twist-ER cells by tamoxifen (GSE139074). (**J**) EMT status in HNF-1β-deficient mIMCD3 cell lines (GSE97770). *: *p* <0.05 for Students’ *t*-test.

Next, among these 77 datasets, we investigated several individual datasets where the EMT/MET phenomenon was induced in different tissues and cell lines. We found that the Py2T murine epithelial tumor cells that exhibit reversible EMT upon treatment with TGF-β in vitro had lower KS and MLR scores but higher 76GS scores as compared to the MTΔEcad cells that represent irreversible EMT murine mammary gland tumor cells (Figure 2A; GSE118612) [44]. Thus, all three EMT scores captured the expected trend of Py2T cells being more epithelial relative to MTΔEcad (murine breast cancer cells with ablated E-cadherin). Further, in mammary epithelial cells (MCF10A), the depletion of Runx1 results in striking morphological changes consistent with EMT [45]. Consistently, Runx1 depleted MCF10A cells had higher KS and MLR scores, but lower 76GS scores (Figure 2B; GSE85857). Similarly, TGF-β-treated primary airway epithelial cells as well as TGF-β- and EGF-treated HeLa cells had a more mesenchymal profile as assessed by 76GS, KS and MLR scores, consistent with their reported experimental trends (Figure 2C,D; GSE72419, GSE61220) [46,47]. These scores were also able to recapitulate in vitro observations that, while TGF-β treatment was able to induce EMT in MCF10A cells, the extent of EMT induced was decreased upon the knockdown of ZEB1 (Figure 2E, GSE1248423) [48], a key EMT-inducing transcription factor in many cancers [49]. ZEB1 forms a mutually inhibitory feedback loop with GRHL2, a crucial MET-inducing factor, and the knockdown of GRHL2 is known to push epithelial or hybrid E/M cells into a more mesenchymal phenotype [50,51,52,53]. Therefore, the OVCA429 cells with GRHL2 knockdown had higher MLR and KS scores, but reduced 76GS scores, as compared to the control, reflective of their more mesenchymal status (Figure 2F, GSE118407) [54]. Similarly, *Grhl2*-null embryos had reduced levels of other gatekeepers of the epithelial phenotype (*Ovol1*, *Ovol2,* and miR-200 family [52,55,56]) and elevated levels of *Zeb1*, commensurate with their altered KS, 76GS, and MLR scores (Figure 2G, GSE106130) [57]. ZEB1 is directly activated by Twist [58], another well-characterized EMT inducer [59]. Thus, the activation of Twist in HMLE (human mammary gland epithelial cells) corresponded to higher KS and MLR scores and reduced 76GS scores (Figure 2I; GSE139074) [60].

Similarly, these scoring metrics could recapitulate the activation of EMT in pre-malignant immortalized and Ras-transformed HMECs (human mammary epithelial cells) as compared to primary HMECs (GSE110677; Figure 2H). Finally, in the context of renal fibrosis caused by the loss of HNF-1β [61], HNF-1β deficient renal epithelial cells mIMCD3 showed upregulated mesenchymal traits relative to wild-type cells, as again captured by the KS, 76GS, and MLR scores (Figure 2J; GSE97770) (Table S2). Together, these case studies demonstrate that each scoring metric can capture the extent of EMT induced upon various perturbations, consistent with the enrichment of EMT depicted by the Hallmark EMT geneset in MSigDB (Molecular Signature Database) (Figure S3) portal [62], in various cancer types as well as in human and mouse cancer cells.

### 3.2. Single-Cell RNA-Seq Data Analysis Reveals Heterogeneity along the EMP Spectrum

**Figure 3:**
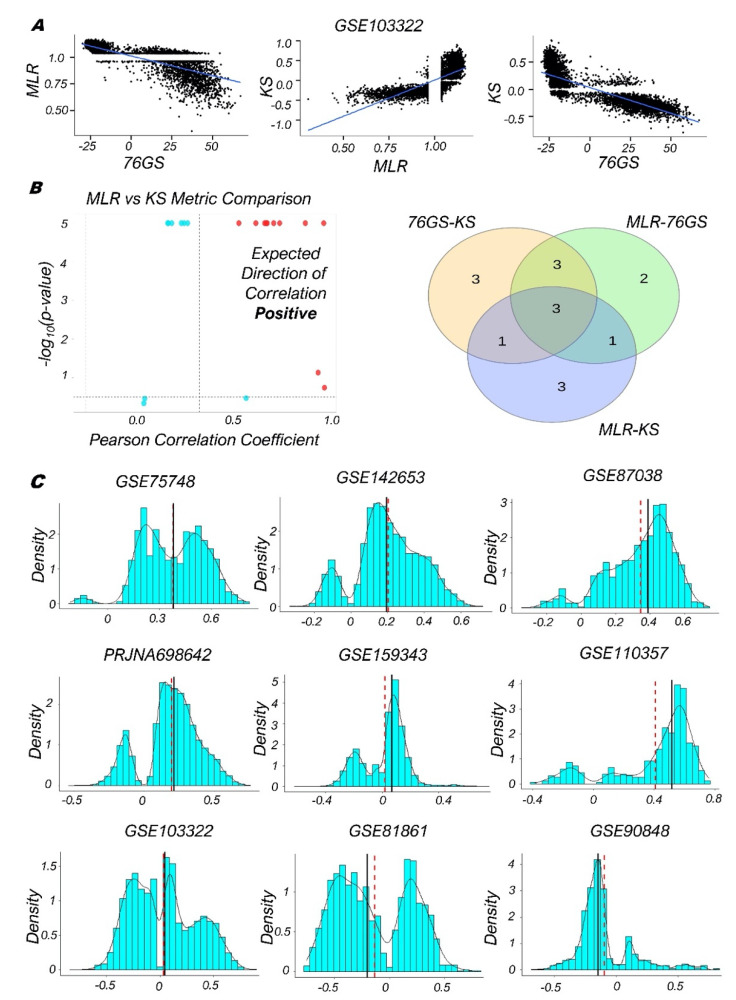
Concordance across EMT scoring methods in single cell RNA-Seq datasets. (**A**) Scatter plot of pairwise correlation estimated by linear regression (blue) in GSE103322 dataset. (**B**) (Left) Plots showing the correlation of MLR vs. KS EMT scoring metrics for different single-cell RNA-Seq datasets, for each sample −log_10_ (*p*-value) is plotted as a function of Pearson’s correlation coefficient. Thresholds for correlation (*R* < −0.3 (green points) or *R* > 0.3 (red points); vertical dashed grey lines) and *p*-values (*p* < 0.05; horizontal dashed grey lines) are indicated. Cyan points denote the cases for which R > −0.3 or R < 0.3. (Right) Venn diagram depicting the common datasets across three pairwise comparisons that are significantly correlated in the expected direction. (**C**) Histograms (teal bars) and corresponding spline interpolate density function estimates (black curves); however, the MLR scores show opposite trends than expected of KS scores in various datasets. Red dashed line and black solid line depicts mean and median, respectively.

After investigating bulk RNA-seq datasets, we calculated EMT scores for 17 single-cell RNA-seq datasets using 76GS, KS, and MLR metrics. For example, in a dataset containing 5902 single cells isolated from 18 patients with oral cavity tumors (head and neck squamous cell carcinoma), we observed a negative correlation between 76GS and KS scores, and between 76GS and MLR ones, with, however, a positive correlation between the KS and MLR scores (Figure 3A; GSE103322) [17]. This trend was largely seen across other single-cell RNA-seq datasets as well, where, similar to our previous results for bulk RNA-seq datasets, roughly 65% (11/17) of datasets showed a negative correlation for 76GS vs. KS scores, 59% (10/17) of datasets had a negative correlation for MLR vs. 76GS, and 53% (9/17) exhibited a positive correlation for MLR vs. KS (Figure 3B,C and Figure S4A). Thus, the concordant trends observed for these metrics using bulk transcriptomics were found to be conserved for single-cell RNA-seq datasets as well (Table S3).

Next, we plotted the histograms for the EMT scores of various single-cell RNA-seq datasets to decipher the heterogeneity seen along the EMP spectrum across a variety of biological contexts: 1. human embryonic stem-cell-derived progenitor cells differentiating to endoderm (GSE75748) [63]; 2. human fetal pituitary gland development including progenitors of many endocrine cell types and subtypes (GSE142653) [64]; 3. cells from different tissues and organs of E9.5 to E11.5 mouse embryos (GSE87038) [65]; 4. MCF10A cells treated with TGF-β for varying durations and exhibiting a gradual change in their EMT status (PRJNA698642) [66]; 5. murine pancreatic duct cells with variations along the EMP spectrum (GSE159343) [67]; 6. EpCAM+ and EpCAM- squamous skin carcinoma cells with varied epithelial and/or mesenchymal features (GSE110357) [68]; 7. cells from oral cavity tumors/head and neck squamous cell carcinoma (GSE103322) [17]; 8. human colorectal cancer cell lines and tumors (GSE81861) [69], and 9. mouse hair follicle stem cells and transit-amplifying cells (GSE90848) [70]. Across these cases, we observed two distinct peaks in KS scoring metrics (Figure 3C), suggesting the presence of at least two major subpopulations with varied EMT status.

Of note, several plots for the 76GS and MLR metrics appeared saturated, which we hypothesized related to the relative sparsity of the predictor signal in the single cell datasets. For the MLR approach, we then restricted our analysis to datasets with at least 90% of all single-cell samples containing nonzero entries for each predictor, indicating the presence of measurable signal. In these cases, MLR and 76GS metrics were able to recapitulate the trends observed in KS for many such datasets (Figure S5A,B).

### 3.3. Quantifying the EMP Spectrum during Lung Diseases and Cellular Reprogramming

All of the three EMT metrics (76GS, KS, MLR) have been designed and/or trained for quantifying the EMT status in cancer samples [30,32,33], but our single-cell RNA-seq data analysis suggests their applicability in various developmental contexts. Thus, we investigated if they can be broadly applied to quantifying the EMT status in biological processes other than cancer. For non-cancer biological contexts, we had not previously looked at EMT score calculations in either microarray or RNA-Seq datasets; thus, for both microarray and RNA-seq datasets (Table S4), we used these metrics to calculate the EMT status for lung diseases including chronic obstructive pulmonary disease (COPD) and idiopathic pulmonary fibrosis (IPF) where EMT is reported to be involved in initiating and/or aggravating the disease [35].

**Figure 4:**
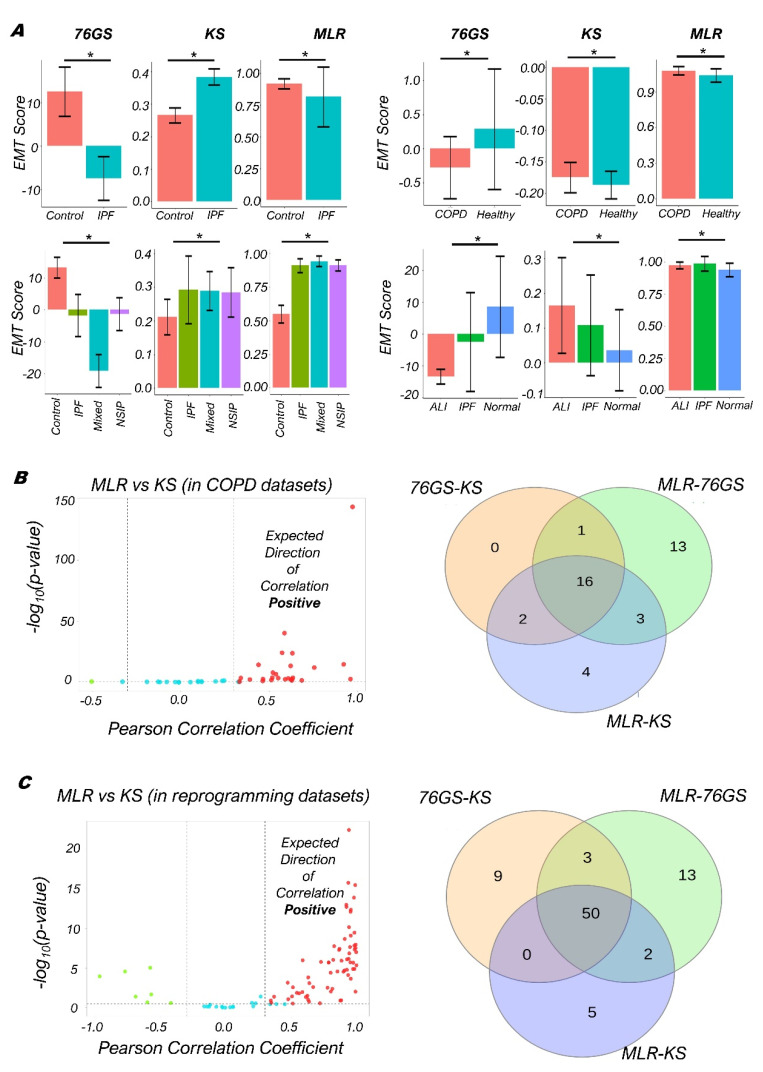
Concordance across all three EMT scoring methods in COPD/IPF and reprogramming datasets. (**A**) Bar plots showing EMT scores of different datasets calculated using three EMT scoring methods—(i) Lung tissues of healthy cases and IPF patients (GSE72073), (ii) EMT status in BAL cells of healthy volunteers and COPD patients (GSE73395), (iii) Lung tissues of IPF, NSIP, and Mixed IPF/NSIP patients, as well as healthy lung tissue (GSE110147), (iv) EMT status in lung tissues of Normal, ALI, and IPF patients (GSE134692). (**B**) (Left) Plots of the correlation of MLR vs. KS EMT scoring metrics across 46 datasets. For each sample, −log10 (*p*-value) is plotted as a function of Pearson’s correlation coefficient. Thresholds for correlation (*R* < −0.3 or *R* > 0.3; vertical dashed grey lines) and *p*-values (*p* < 0.05; horizontal dashed grey lines) are shown. (Right) Venn diagram depicting the common datasets across all pairwise comparisons that are significantly correlated in the expected direction. (**C**) Same as (**B**) but for reprogramming associated datasets. *: *p* < 0.05 for Students’ *t*-test.

As compared to normal lung tissues, the fibrotic lung tissues from IPF patients had higher KS scores but lower 76GS scores, indicating their enhanced mesenchymal status (Figure 4A, i; GSE72073); however, the MLR scores show opposite trends than expected. Fibrotic lung tissues had reduced levels of USP13, a deubiquitylase that stabilizes PTEN, and in vitro analysis suggested that USP13 deficiency increased the invasive and migratory capacities of fibroblasts, traits usually associated with EMT [71]. Similarly, relative to healthy volunteers, COPD patients showed increased EMT in their bronchoalveolar lavage (BAL) cells (Figure 4A, ii; GSE73395) [72]. Consistently, as compared to normal lung tissue, patients with any of the three lung pathological situations—IPF, non-specific interstitial pneumonia (NSIP) and mixed IPF-NSIP—exhibited trends of enhanced EMT (Figure 4A, iii; GSE110147) [73]. Further, RNA-seq analysis of lung tissues of patients with acute lung injury (ALI) and IPF had higher MLR and KS scores but reduced 76GS scores (Figure 4A, iv; GSE134692), consistent with earlier reports [74,75,76].

After investigating these few examples, we analyzed the trends among the KS, MLR, and 76GS scores obtained for 46 microarray or RNA-seq datasets associated with lung injury. Reinforcing the trends seen for cancer-related datasets, 76GS and MLR scores were negatively correlated in roughly 72% (33/46) of datasets. Similarly, 76GS and KS scores correlated negatively in ~41% (19/46) of datasets. Further, KS and MLR scores correlated positively in ~54% (25/46) of datasets (Figure 4B and Figure S4B). Overall, we see a strong concordance among the three EMT scoring metrics for non-cancerous lung diseases too. Thus, for patients suffering from IPF, COPD, or lung injury, tracking their EMT status can help identify the degree of progression of the disease.

Further, we investigated a set of datasets related to the cellular reprogramming of differentiated cell types to induced pluripotent stem cells (iPSCs), where EMT/MET are reportedly involved [37] (Table S5). Across the 92 datasets for which we calculated the 76GS, KS, and MLR EMT scores, roughly 62% (57/92) showed a positive correlation between MLR and KS, while 67% (62/92) showed a negative correlation between KS and 76GS, and approximately 74% (68/92) showed a negative correlation between the corresponding 76GS and MLR ones. Overall, 54% (50/92) of datasets demonstrated all three pairwise correlations to be strong (Figure 4C and Figure S4C), thus endorsing that these EMT scoring metrics can be quite consistent with one another in terms of identifying the EMP status of cells en route to cellular reprogramming.

### 3.4. Context-Specific Association of EMP Status with Patient Survival

**Figure 5:**
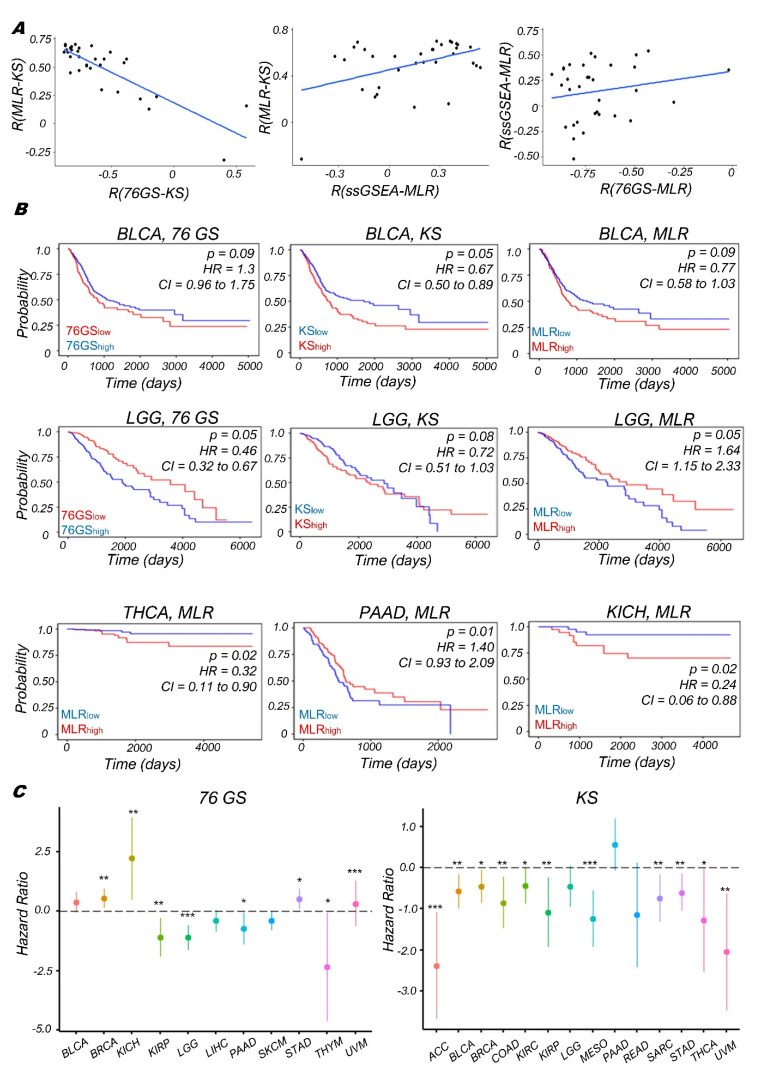
EMT scoring in TCGA datasets and survival analysis using patient samples. (**A**) Scatter plot of pairwise correlation estimated using linear regression (blue) in TCGA datasets. (**B**) Correlation between EMT score (high vs. low) and overall survival (OS) in various TCGA datasets. Kaplan–Meier survival analysis is performed to estimate differences in survival for different EMT metrics. *p*-values (*p*) reported are based on log rank test. The values of Hazard ratio (HR) with 95% confidence interval (CI) values are included. (**C**) Plot of log2 hazard ratio (HR; mean ± 95% confidence interval) comparing overall survival (OS) of 76GS (left) and KS (right) EMT scoring metrics on different TCGA cancers and cohorts. *p*-values are based on log-rank test, and those with significant differences (*p* < 0.05) are marked with a star (*); **: *p* < 0.01; ***: *p* < 0.001.

Next, we quantified the EMT scores in patient samples using TCGA datasets of various cancer types. Here, also we found the expected trends that the 76GS scores show negative correlation with the MLR and KS scores and KS and MLR scores are positively correlated to each other (Figure 5A), reinforcing our observations for a pan-cancer analysis of microarray datasets (Table S6) [77]. We also calculated Single-set Gene Set Enrichment Analysis (ssGSEA scores) [78] using the EMT gene set from MSigDB [62]. Each ssGSEA enrichment score represents the degree to which the genes in a particular gene set are coordinately regulated within a sample. We find that the ssGSEA scores for EMT show, as expected, a negative correlation with the 76GS scores and a positive correlation with the MLR and KS scores.

We also assessed the association between the EMT scores and patient survival using different survival data types (Overall survival (OS), Disease-Specific Survival (DSS), Progression free interval (PFI), and Disease-free interval (DFI)) in various TCGA cancer cohorts. The samples were scored using all three methods and segregated into high and low groups based on the mean value of each EMT score. The 76GS^low^ subgroup can be thought of as comparable to the MLR^high^ and KS^high^ groups, given their relatively strong M signature. In bladder cancer (BLCA; Figure 5B, top), we see consistent trends in the case of overall survival (OS) for all three types of EMT scores that the stronger the M phenotype is, the worse the survival probability is; whereas in the case of Low-Grade Glioma (LGG), we see the opposite trend, that is, the stronger the M phenotype is, the better the survival probability is (Figure 5B, middle). Similarly, in Thyroid Cancer (THCA) and Kidney Chromophobe (KICH), higher MLR scores (a more M phenotype) reflect worse survival outcomes, but in pancreatic adenocarcinoma (PAAD), they associate with better outcomes (Figure 5B, bottom), indicating a context-specific association of the extent of EMT with patient survival. A pan-cancer analysis reveals further evidence for this context-specific behavior; hazard ratio (HR) > 1 and HR < 1 scenarios were both observed depending on the cancer subtype in TCGA (Figure 5C and Figure S6). For instance, in breast cancer (BRCA), stomach adenocarcinoma (STAD), and in uveal melanoma (UVM), similar to BLCA, stronger M phenotypes (as identified by KS^high^ or 76GS^low^) corresponded to worse overall survival. However, similar to PAAD, for the cases of kidney renal papillary cell carcinoma (KIRP), thymoma (THYM), and low-grade glioma (LGG), the stronger the M phenotype is (as identified by 76GS^low^), the better the survival is. 

While the concept of EMP may not as be as stringently applicable to non-epithelial cancers such as LGG, these pan-cancer observations highlight the following two key aspects: (a) the categorization of patient samples with more vs. less M phenotypes is largely consistent across the three scoring metrics used, and (b) the often-assumed association of EMT with worse survival is not a universal feature; instead, it depends on the specific cancer subtype.

**Figure 6:**
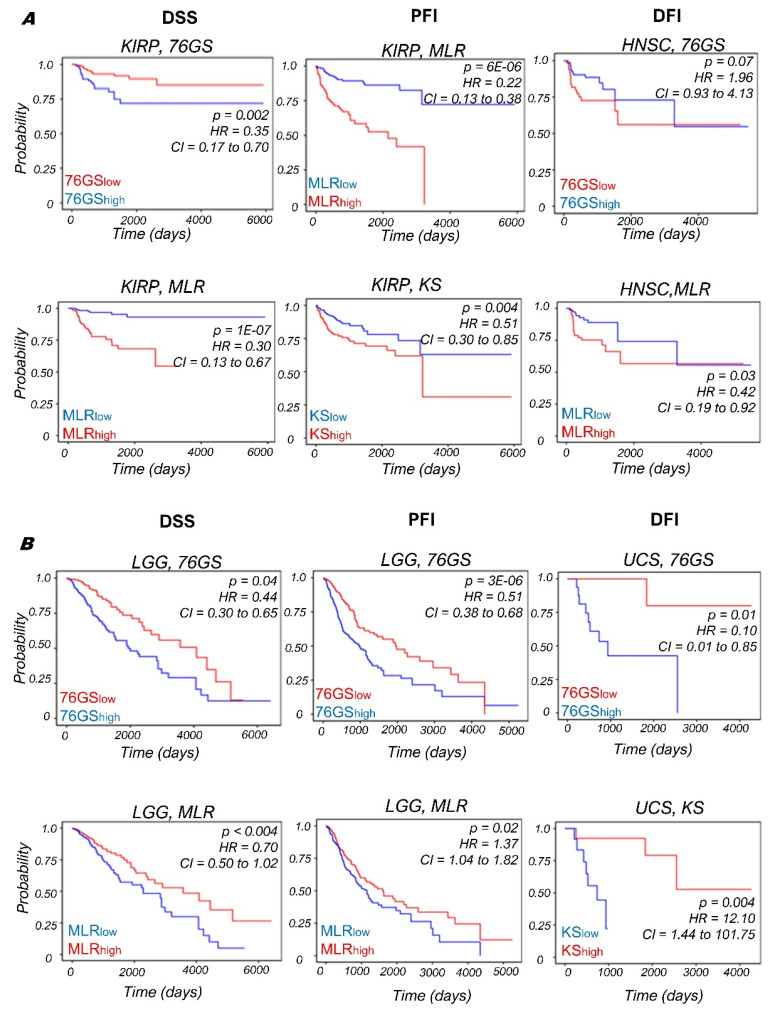
Correlation between EMT score (high vs. low) and various survival types (DSS, PFI, and DFI) in various TCGA datasets. Kaplan–Meier survival analysis is performed to estimate differences in survival for different EMT metrics. *p*-values (*p*) reported are based on the log rank test. The values of Hazard ratio (HR) with 95% confidence interval (CI) values are included. (**A**) Plots for (kidney renal papillary cell carcinoma (KIRP) and Head and Neck Squamous Cancer (HNSC). (**B**) Plots for low-grade glioma (LGG) and uterine carcinosarcoma (UCS).

After investigating the OS data, we calculated the survival probabilities through other metrics as well—DSS, PFI and DFI, wherever available. For DSS, we found that the KIRP samples with a stronger M phenotype (as identified by MLR^high^ or 76GS^low^) reflect poorer survival. This trend held for PFI as well (Figure 6A; columns 1, 2); however, this is in contrast to the observations for KIRP for OS earlier. On the other hand, consistent with observations made for OS, for LGG samples, an improved DSS and PFI was associated with a stronger M phenotype (as identified by MLR^high^ or 76GS^low^) (Figure 6B; columns 1, 2). The DFI for Head and Neck Squamous Cancer (HNSC) indicates a worse prognosis associated with a stronger M phenotype (as identified by MLR^high^ or 76GS^low^) (Figure 6A; column 3), opposite to that seen for the case of Uterine Carcinosarcoma (UCS) (Figure 6B; column 3), indicating that the UCS samples with enriched M phenotypes correspond to improved disease-free survival. Together, the context-specific association of EMT with patient outcome in cancer types is visible across many clinical metrics—OS, DSS and PFI, suggesting caution in categorizing patients based on EMT for any treatment strategies (Table S7).

## 4. Discussion

Quantifying the spectrum of epithelial-hybrid-mesenchymal cell states in cancer has garnered recent interest due to a surge in the availability of in vitro and in vivo spatial and/or temporal dynamic and high-throughput data at multiple levels—transcriptomic, proteomic, epigenetic, metabolic, and morphological [66,79,80,81,82,83,84,85,86,87,88]. Phenotypic plasticity and heterogeneity along the EMP spectrum have been postulated to be a more important criteria for defining the survival fitness of a cancer cell population than the predominance of a specific phenotype [89,90], suggesting possible benefits to a more heterogeneous population through cooperation among cancer cells with varying EMP phenotypes [91,92]. For single cells, hybrid E/M phenotypes are believed to be the most plastic relative to their more “extreme” epithelial and mesenchymal counterparts; such plasticity can amplify the tumor-initiation ability [18,93]. Therefore, characterizing the EMP as a continuous spectrum instead of as an “all-or-none” process becomes imperative for an improved understanding of the emergent dynamics of EMT and MET, and their relevance to patient survival.

Here, we used three different EMT transcriptomic-based scoring metrics, each of which was developed using cancer cell lines and/or tumor samples and tested on microarray data previously to quantify the extent of EMT in a continuum—76GS, KS, MLR. Using these metrics, we calculated EMT scores for over 100 RNA-seq datasets—including at bulk and single-cell levels—across multiple biological contexts (cancer (Figure 2 and Figure 3), fibrosis, COPD, and cellular reprogramming to iPSC (Figure 4)). We observed that these methods show a high degree of concordance among themselves in their ability to identify the extent of EMT/MET a sample has undergone, despite using different gene lists and algorithms (Table S8). This concordance suggests an overlap of the core expression patterns central to EMT in a high-dimensional feature space and indicates that these metrics—initially developed for cancer samples—can be applied more generally to a broader range of biological contexts.

Using these metrics in biological contexts where hybrid E/M states have been proposed [35,94,95] may be helpful in mapping the corresponding trajectories of EMT/MET. The hybrid E/M state has been previously documented at both bulk and single-cell levels during various stages of development as well [65,96]; here, we have shown proof of the principle that these scoring metrics can successfully quantify the extent of EMP in mouse models. We are, however, not suggesting that any one particular method is better than the others. These scores are calculated using three different gene sets and different algorithms and still shows concordance in various biological contexts. Our results detail the concordance amongst the three methods on many of the testing examples, and in doing so, illustrate the context-specific strengths of each method. However, whether they can be adapted to adequately investigate the role of EMT in applications for other non-human model organisms remains to be investigated. Another direction of future work includes that given sufficient patient data, the EMT spectrum quantification within a given cancer subtype can provide an early forecast for disease aggressiveness based on historically aggressive EMT signatures.

In our analysis of single-cell RNA-seq data, the resolvability of bimodal distributions consistent with dual sub-populations was optimally characterized using the KS scoring metric. Improvements in the MLR and 76GS approaches were observed when restricting the analysis to datasets with non-zero MLR predictors for a majority (>90%) of the single-cell samples. These scores, while concentrated in the middle of the EMT interval, were able to recover features of the distributions observed via the KS method. Together, this suggests that the development of optimized signal-to-noise criteria, may improve the absolute placement of samples on the EMT MLR spectrum and is the focus of our ongoing research effort, building upon various tools used in the analysis presented here (Table S9). Future efforts should also consider how these metrics can be adapted to investigate different cell-state transition trajectories, for instance, by defining a two-dimensional (2D) EMT score that can deconvolute gains in the mesenchymal program vs. losses in the epithelial one [97]. The three EMT scoring metrics have been helpful in investigating the association of EMT/MET with other aspects of cellular plasticity such as stemness [98], immune evasion [99], and sensitivity to anti-cancer agents [100]. However, future work remains to be conducted on how to connect these data-based metrics with insights from mechanism-based dynamical models of EMT [101,102]. For instance, these data-based metrics can be used to validate whether EMT and MET follow similar trajectories in a high-dimensional landscape; recent transcriptomic and proteomic experiments suggest hysteresis [87,103], as has been identified from mechanism-based models [102].

Intriguingly, the EMT status of primary tumors was not found to be universally correlated with worse patient survival, but instead showed a context-dependent trend (Figure 5 and Figure 6), consistent with previous reports [32]. EMP is a highly dynamic trait. Thus, capturing static snapshots of gene expression profiles may not be sufficient for recapitulating the dynamic dependence of cancer cell fitness on EMT and/or MET. Thus, the EMT status and/or heterogeneity of a primary tumor may not reflect that of circulating tumor cells (CTCs) and their metastatic potential, leading to such observed context-specific trends. Moreover, transcriptomic profiles may not be sufficient to indicate phenotypic variability and incorporate epigenetic and/or metabolic status can elucidate the manifestations of dynamic adaptation during metastasis. Understanding the interplay among EMT, metabolic and epigenetic reprogramming [82,84,104,105,106,107,108] will be key for better patient stratification and therapeutic strategies.

## Data Availability

All codes used in the manuscript are given at https://github.com/sushimndl/EMT_Scoring_RNASeq (accessed on 21 December 2021).

## Supplementary Materials

The following supporting information can be downloaded at: https://www.mdpi.com/article/10.3390/biom12010029/s1, Figure S1: Linear regression on log2 FPKM-normalized RNA-seq and log2 microarray correlation plots; Figure S2: Linear regression on log2 TPM-normalized RNA-seq and log2 microarray correlation plots; Figure S3: Bar plots showing ssGSEA scores of different datasets (ref Figure 2) calculated using EMT gene set from MSigDB; Figure S4: Concordance among KS, 76GS, and MLR metrics in multiple biological contexts; Figure S5: Heterogeneity in EMP; Figure S6: Kaplan–Meier analysis. Table S1: Details of 77 bulk RNA-seq datasets (GSE ID, PMID, no. of samples, and pairwise correlation coefficient and p-values); Table S2: Details of sample IDs in individual datasets that have been considered for comparative analysis in Figure 2; Table S3: Details of 19 single-cell RNA-seq datasets (GSE ID, PMID, no. of samples, and pairwise correlation coefficient and *p*-values); Table S4: Details of 46 RNA-seq and microarray datasets pertaining to COPD, IPF, and other lung diseases (GSE ID, PMID, no. of samples, and pairwise correlation coefficient and *p*-values); Table S5: Details of 92 RNA-seq and microarray datasets pertaining to cellular reprogramming (GSE ID, PMID, no. of samples, and pairwise correlation coefficient and *p*-values); Table S6: Details of datasets included in EMT-ome [77] together with pairwise correlation coefficient and *p*-values among EMT scoring metrics; Table S7: Details of survival analysis of cancer types from TCGA cohort (OS, DFI, DSS and PFI) containing Hazard Ratio (HR), 95% Confidence interval (CI) and corresponding *p*-values. Red suggests M is worse and Blue suggests E is worse. Dark color represents *p* < 0.05; light color denotes *p* < 0.1. Table S8: List of genes common across the three scoring metrics. Table S9: List of software used in this study.
