# Supplementary figures and images for: Transcriptomic-Based Quantification of the Epithelial-Hybrid-Mesenchymal Spectrum across Biological Contexts
Source: Biomolecules. 2021 Dec 25;12(1):29. doi: 10.3390/biom12010029 (PMC8773604; doi:10.3390/biom12010029)

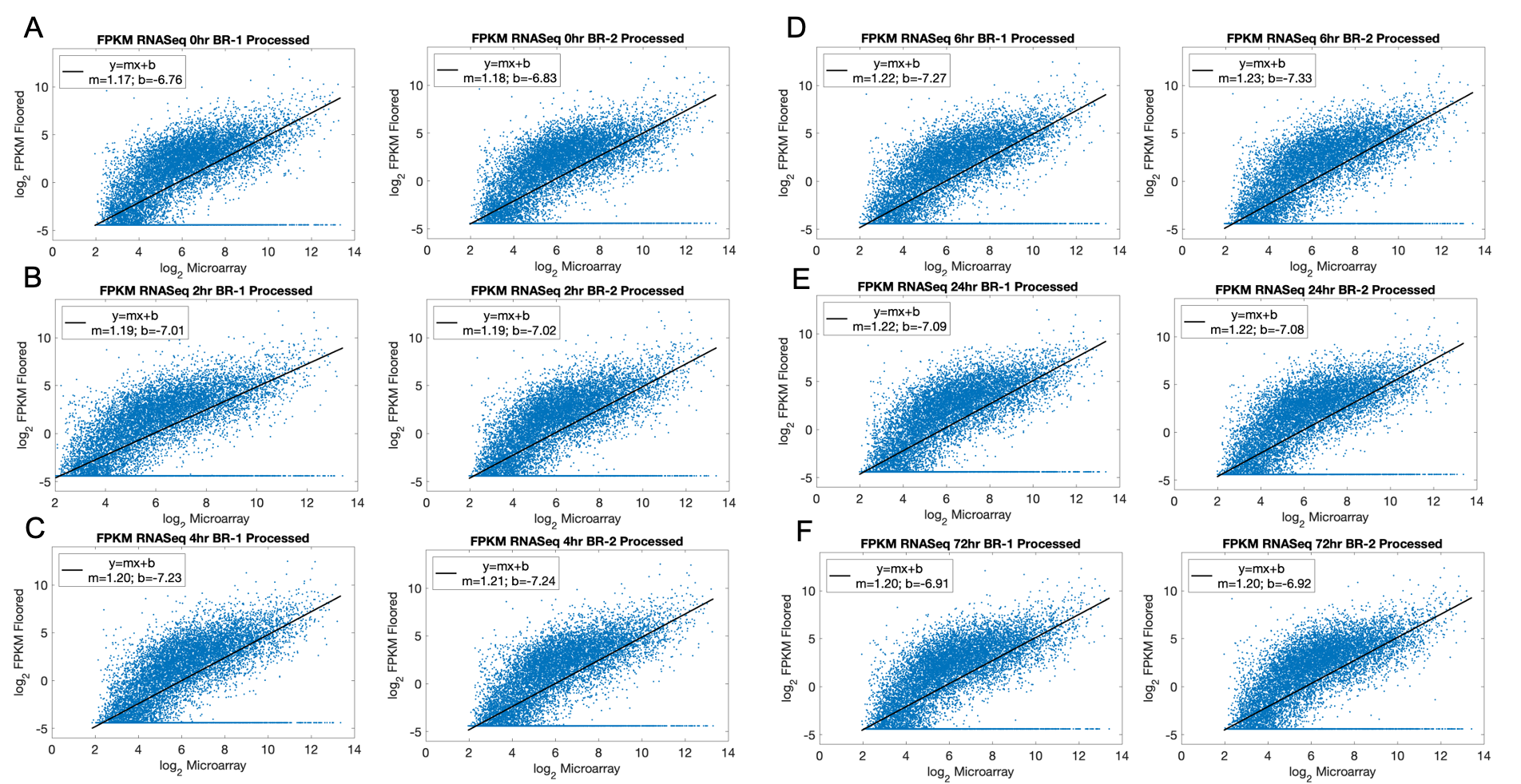

Supplement: Supplementary file 1 [file biomolecules-12-00029-s001.zip › Fig S1_MLR.png]

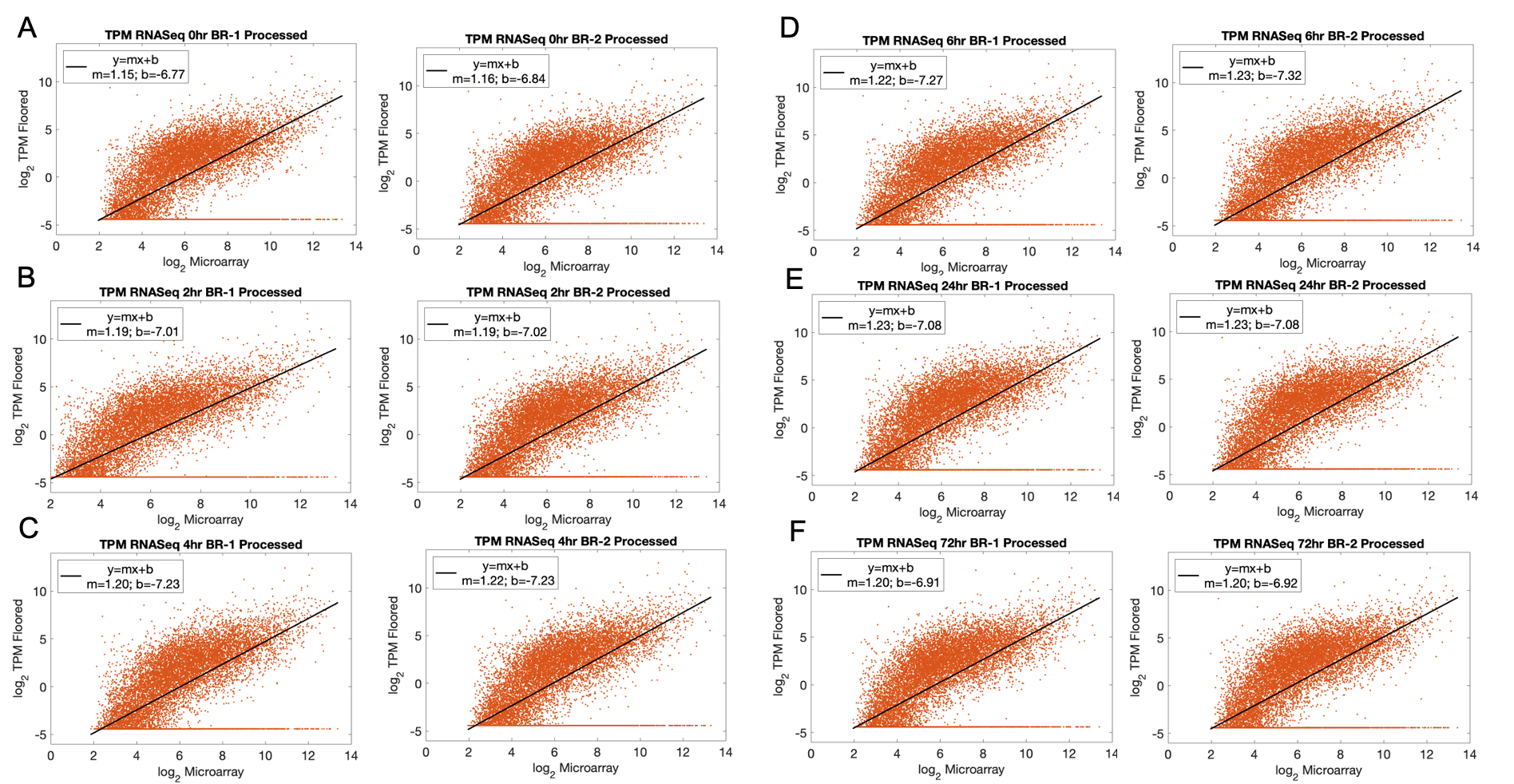

Supplement: Supplementary file 1 [file biomolecules-12-00029-s001.zip › Fig S2_MLR.png]

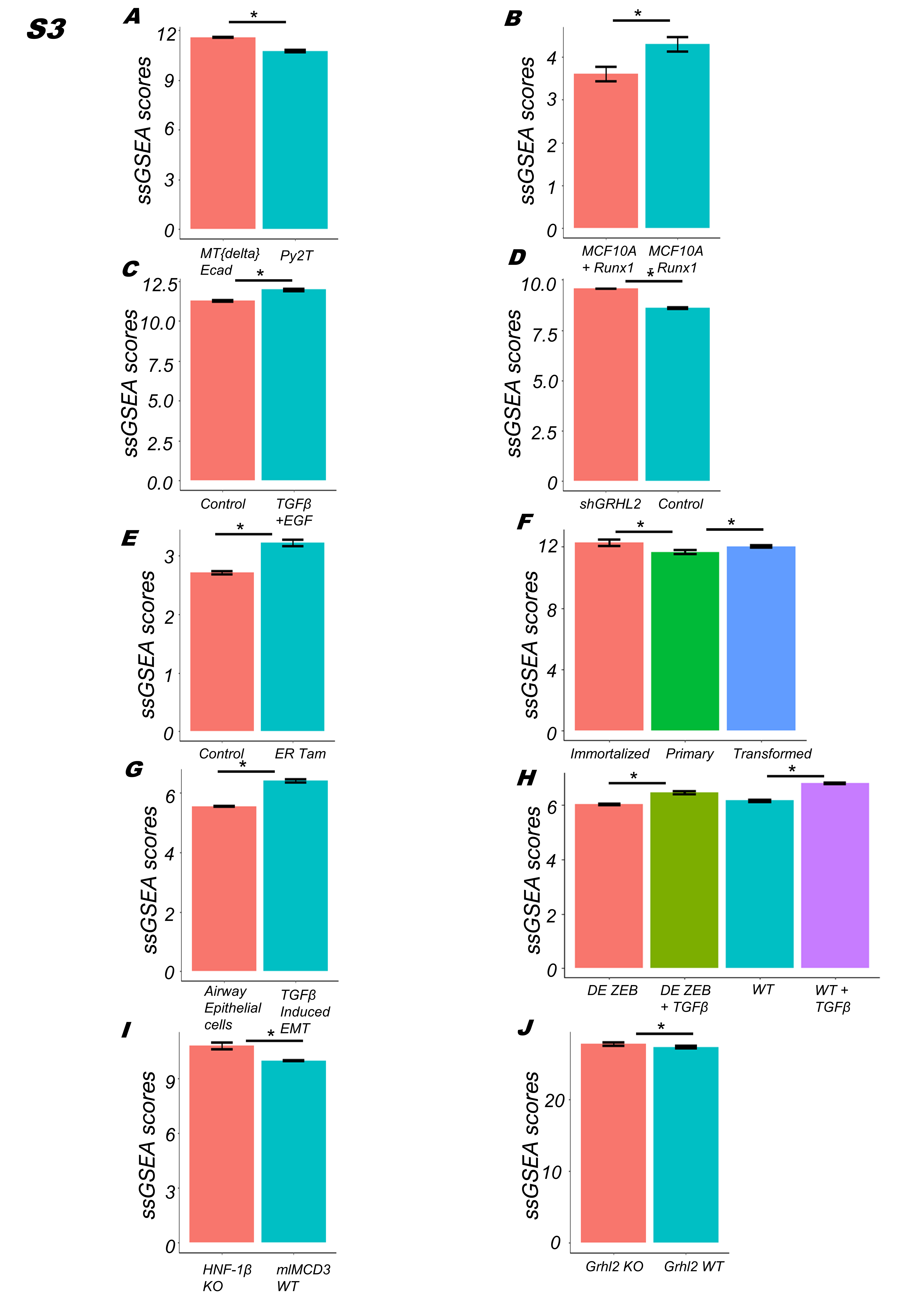

Supplement: Supplementary file 1 [file biomolecules-12-00029-s001.zip › Fig S3_MLR.PNG]

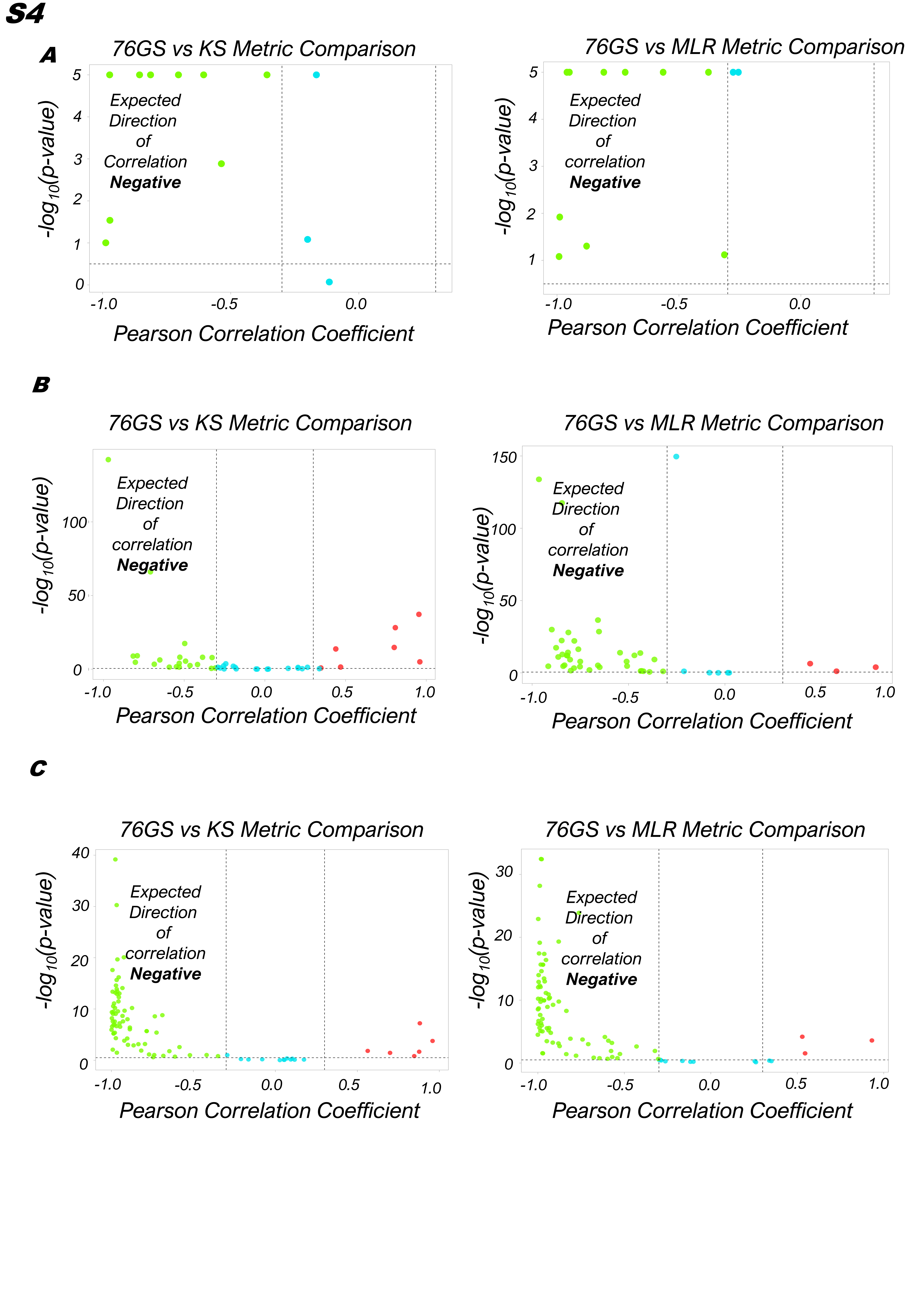

Supplement: Supplementary file 1 [file biomolecules-12-00029-s001.zip › Fig S4_MLR.PNG]

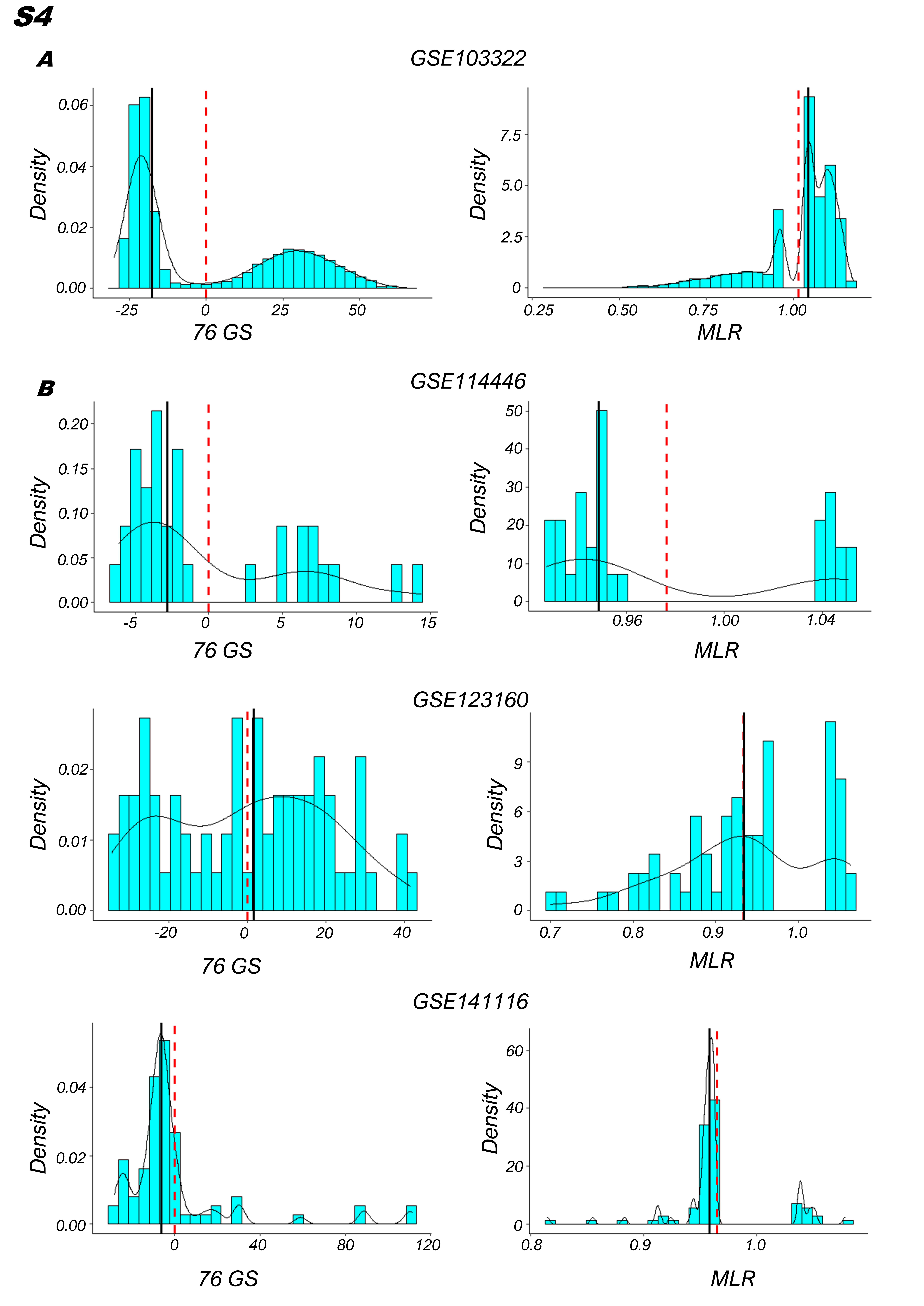

Supplement: Supplementary file 1 [file biomolecules-12-00029-s001.zip › Fig S5_MLR.PNG]

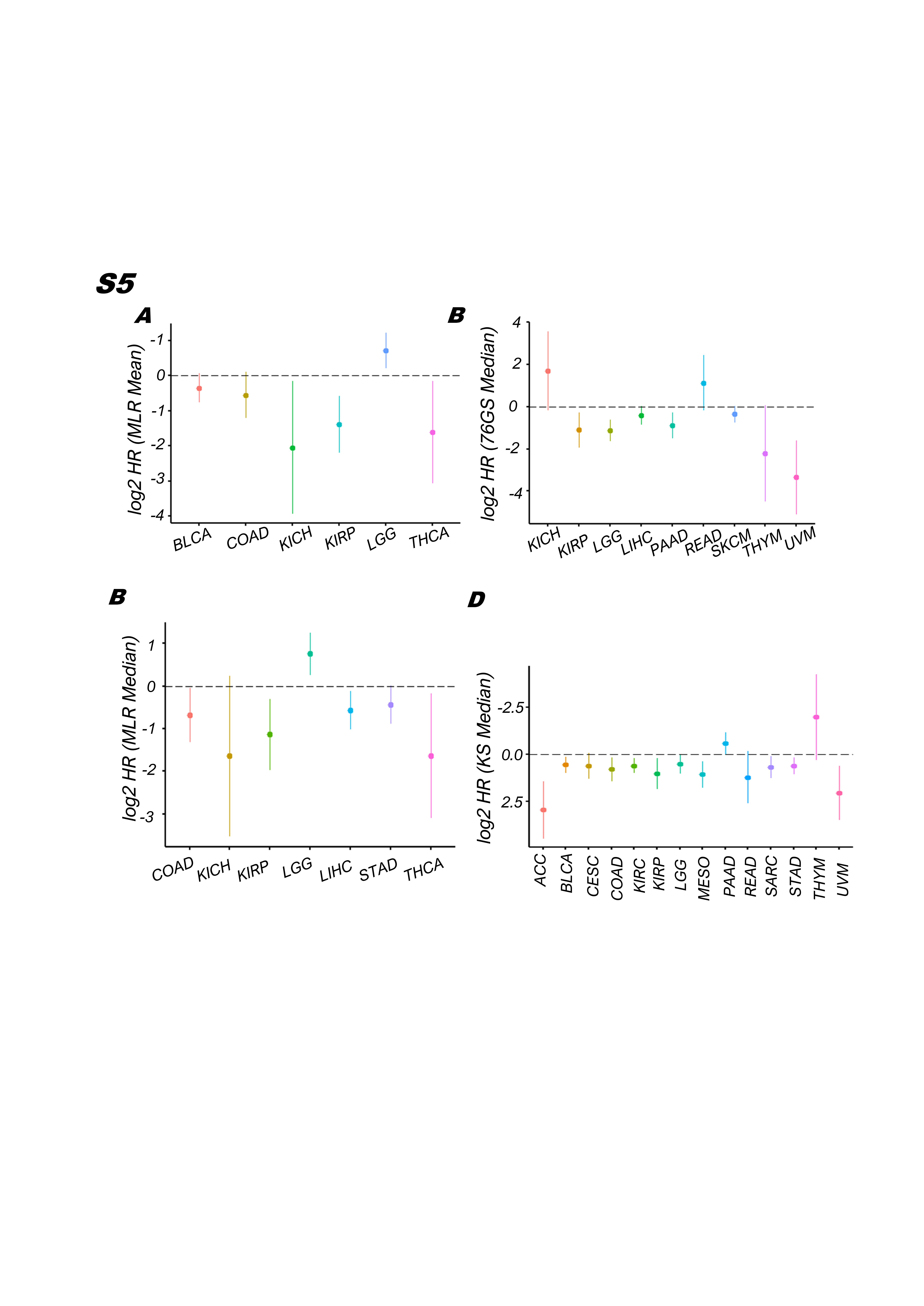

Supplement: Supplementary file 1 [file biomolecules-12-00029-s001.zip › Fig S6_MLR.PNG]
